# Associations of Sleep Quality, Anxiety, and Depression with Cognitive and Executive Functions among Community-Dwelling Women Aged ≥ 65 Years: A Cross-Sectional Study

**DOI:** 10.3390/healthcare9111599

**Published:** 2021-11-21

**Authors:** Ana Belén Parra-Díaz, Agustín Aibar-Almazán, Antonio Martínez-Amat, José Daniel Jiménez-García, Francisco Álvarez-Salvago, Fidel Hita-Contreras

**Affiliations:** 1 Department of Health Sciences, Faculty of Health Sciences, University of Jaén, 23071 Jaén, Spain; abpd0002@red.ujaen.es (A.B.P.-D.); amamat@ujaen.es (A.M.-A.); josedanieljimenezgarcia@gmail.com (J.D.J.-G.); fhita@ujaen.es (F.H.-C.); 2 Department of Physiotherapy, Faculty of Health Sciences, European University of Valencia, 46112 Valencia, Spain; salvagofran@gmail.com

**Keywords:** cognitive performance, verbal fluency, executive function, sleep quality, anxiety, depression, women

## Abstract

(1) Background: The objective of this study was to evaluate the associations of sleep quality, anxiety, and depression with cognitive performance, executive functions, and verbal fluency among women aged ≥ 65 years; (2) Methods: A cross-sectional study was conducted on 241 women (72.52 ± 3.93 years). Cognitive performance (Mini-Mental State Examination) and impairment (Montreal Cognitive Assessment), verbal fluency (Isaacs test) and executive function (Trail Making Test), Sleep quality (Pittsburgh Sleep quality Index) and anxiety and depression (Hospital Anxiety and Depression Scale) were determined; (3) Results: The linear regression analysis indicated that anxiety, depression and age, were related to lower Mini-Mental State Examination score (adjusted R^2^ = 0.306), and age, anxiety and daytime dysfunction were linked to reduced Montreal Cognitive Assessment score (adjusted R^2^ = 0.248). Age and daytime dysfunction were associated with worse verbal fluency (adjusted R^2^ = 0.094). Finally, sleep latency, sleep disturbances, the Pittsburgh Sleep quality Index total score were associated with longer times in TMT-A (adjusted R^2^ = 0.758) and TMT-B (adjusted R^2^ = 0.508); (4) Conclusions: Sleep quality was associated with cognitive performance, verbal fluency and executive functions. Besides, both anxiety and depression were related with cognitive performance, while only anxiety was linked to executive functions. As for confounders, age was associated with cognitive performance and verbal fluency.

## 1. Introduction

Aging is a natural process that is part of people’s lives [1]. The world population is getting older and older due to increased life expectancy and decreased fertility, and it is estimated that, by the middle of the 21st century, the percentage of people over 60 years of age will increase to 22% [1,2]. From the age of 60, the aging process, a series of biological, psychological and social changes, begins to appear [3], which can generate functional limitations in the quality of life of older adults, and this situation can be worsened in older women due to menopause [4]. The menopausal stage constitutes a third of a woman’s life [5], so it is essential to analyze the physical and psychological problems associated with it and thus be able to improve their quality of life. Some of the most common psychological problems in menopause are depression, described as the presence of an emotional state of sadness that is linked to other alterations causing discomfort and changes in the functionality of the person who suffers [6], and anxiety, that is defined as a feeling of fear or fear that can affect cognitive, behavioral and/or somatosensory levels in people [7]. Both occur because productivity, functional capacity and personal self-satisfaction are diminished at this stage of life [8] and are associated with high comorbidity such as cognitive problems, insomnia and physical dysfunction [9,10].

Another of the most frequent problems during menopause is the decrease in the quality of sleep, since sleep is related to organic and psychological changes that occur at this stage [11]. An association between the daily amount of sleep, the quality of sleep and age has been demonstrated [12] and this may be due to attention and concentration difficulties, psychological and social problems and also the multiple pathologies suffered by these people [13]. In this period, postmenopausal women have more awakenings throughout the night, wake up earlier and have fewer reveries in the REM phase [14], which leads to an increase in naps and drowsiness during the day. Then they cause changes in the circadian cycles [13], which causes women to be less emotionally active when carrying out their daily activities, to become more tired and thus unable to concentrate and maintain good attention [13,14].

As people age, people tend to suffer mild cognitive impairment, mainly memory, attention, verbal fluency, and executive functions [15]. These cognitive problems are more aggravated in postmenopausal women due to the reduction of androgens and estradiol, which have a fundamental role in brain neuroprotection, since they collaborate in neuronal growth, prevent its loss and are also synaptic transmitters [16,17]. These hormonal imbalances also often affect the quality of sleep and bring with them emotional problems that can affect cognitive performance [12]. Furthermore, 63% of dementia cases in the world occur in women and are expected to increase due to life expectancy in women [18].

Based on the above, this study aims to analyze the associations of sleep quality, anxiety and depression with cognitive function among community-dwelling older women. We hypothesized that higher levels of anxiety and depression symptoms, as well as poorer sleep quality, are independently associated with worse cognitive performance, executive functions and verbal fluency.

## 2. Materials and Methods

### 2.1. Study Participants and Design

**Figure 1:**
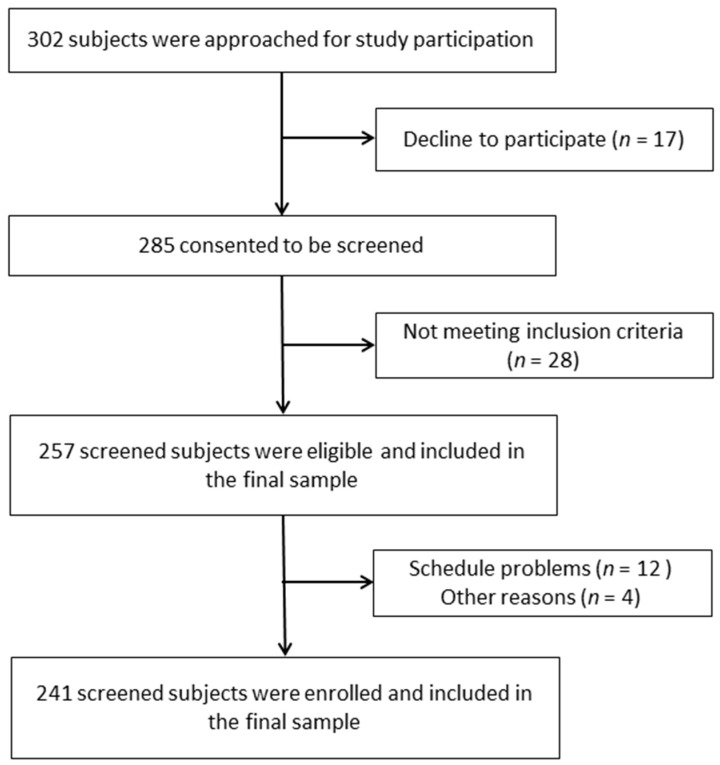
Flow chart of the study participants.

This is an observational cross-sectional study performed to analyze the associations between cognitive function and anxiety, depression and sleep quality in older community-dwelling Spanish women. From a total of 302 women, who were initially contacted, 241 participants were finally included in this study (Figure 1). All the participants were recruited from several senior centers from the Eastern Andalusia region. Each woman firmed an informed consent form before enrollment. This study was approved by the Research Ethics Committee of the University of Jaén, Spain (SEPT.20/2./TES) and was conducted in accordance with the Declaration of Helsinki, good clinical practices, and all applicable laws and regulations. Inclusion criteria: women aged 65 years and over who were able to understand the instructions, programs, and protocols of this study. Exclusion criteria were: women diagnosed with any type of dementia (i.e., Alzheimer, dementia with lewy bodies, or frontotemporal dementia) or other neuropsychiatric diseases, taking sedatives or sleep medication or did not understand the items of the questionnaires.

### 2.2. Outcomes

Sociodemographic data, such as the age and number of years in the menopausal status, education (primary or less/secondary or higher) and smoking habit (yes/no) were collected by face-to-face interviews. Weight and height were assessed by a 100 g–130 kg precision digital weight scale (Tefal) and an adult height scale (T201-T4 Asimed). Body mass index (BMI) [19] was calculated by dividing the woman’s weight (kg) by her height (m^2^). A BMI < 25 kg/m^2^ indicates normal weight, overweight when 25 ≤ BMI > 30 kg/m^2^, and obesity when BMI ≥ 30 kg/m^2^.

#### 2.2.1. Anxiety and Depression

The level of anxiety and depression symptoms was assessed by the Hospital Anxiety and Depression Scale (HADS) [20,21], a well-known instrument employed in the general population. It consists of 14 items of which seven correspond to the anxiety subscale and the other seven to the depression subscale. The scores of each one of the subscales range from 0 to 21, where greater values represent more severe symptoms. A score of ≥11 has been shown to detect cases of both anxiety and depression.

#### 2.2.2. Sleep Quality

The Pittsburgh Sleep Quality Index (PSQI) [22,23] was used to evaluate sleep quality. It consists of 19 items, as well as five more to be answered by bedmates or roommates (these last five items are only used for clinical information). The PSQI provides a total score and seven domain scores ranging from 0 to 3 points, where greater scores indicate poorer sleep quality. These domains are: sleep quality, sleep latency, sleep duration, sleep efficiency, sleep disturbances, use of sleep medication and daytime dysfunction. Total score ranges from 0 to 21, and a score > 5 indicates poor sleep quality [24].

#### 2.2.3. Cognitive Performance

Mini Mental State Examination (MMSE) was used to measure global cognitive function [25]. It is one of the most commonly used tests for the detection of serious cognitive deficiencies, and examines the five areas of cognition: attention, orientation, calculation, registration, language and memory. The maximum score is 30 points, and a higher score indicates a better general cognition of the participant. The reference scores are: dementia: 9 to 12; deterioration: from 12 to 24; pathological suspicion: 24 or less; and normal: 27 or more.

#### 2.2.4. Cognitive Impairment

The Montreal Cognitive Assessment (MoCA) is a short test composed of 12 items that assesses cognitive function through seven cognitive domains: visuospatial and executive function (task B of tracing (1 point), copy of the cube (1 point) and clock drawing (3 points)), denomination (3 points), attention (forward/towards behind), digit interval (2 points), vigilance/tapping (1 point) and subtraction from series (3 points)), language (repetition of sentences (2 points) and verbal fluency (1 point)), abstraction (the element 2-element verbal abstraction, total of 2 points), delayed recall/short-term memory (5 points) and orientation (6 points). The maximum MoCA total score is 30, and values ≥ 26 indicate normal cognitive functioning [26].

#### 2.2.5. Verbal Fluency

In order to measure verbal fluency, the Isaacs test was used. This test consists of the participants reproducing the greatest number of words within a semantic category, such as animals, fruits, cities and colors, for a total of 60s. In each of these categories, the maximum score that can be obtained is 10 points, for a total of 40 points. A higher score indicates a better level of verbal fluency [27].

#### 2.2.6. Executive Functions

The Trail Making Test (TMT) was used for the evaluation of executive function and specifically measures timed motor and visual tasks. This test is divided into two parts: part A (TMT-A) in charge of evaluating psychomotor attention and speed and consists of connecting consecutively numbered circles; and Part B (TMT-B), which is based on connecting alternating circles of letters and numbers and measures executive function [28]. A longer time spent in completing the test was interpreted as a poorer performance.

### 2.3. Sample Size Calculation

For sample size calculation, and according to the recommendations by Concato et al. [29], at least 20 subjects per event are required in the multivariate linear regression model. In this study, eleven events were used: depression and anxiety scores, PSQI total score and its seven PSQI domains, as well as age. Therefore, 220 participants were required for this analysis. The final number of participants was 241.

### 2.4. Data Analysis

Continuous and categorical variables were summarized as means and standard deviations, and frequencies and percentages, respectively. Normality of the data was assessed by the Kolmogorov–Smirnov test. A bivariate and partial correlation analysis and a Student *t* test were performed to evaluate the possible individual associations of anxiety, depression and sleep quality (independent variables) and possible confounders (age, education and smoking habit) with cognitive performance and impairment, verbal fluency and executive function (dependent variables). Then, a multivariate linear regression model, as well as a stepwise method for introducing variables into the model, were used to determine the independent associations. Cognitive performance and impairment, verbal fluency and executive function were individually introduced as dependent variables in separate models. Only those independent variables and possible confounders which showed significant individual associations (*p* < 0.05) were included in the multivariate linear regression. In order to assess the effect size coefficient of multiple determinations in the linear models, adjusted- R^2^ was obtained. Values < 0.02 can be considered as insignificant and small when 0.02–0.15, medium when 0.15–0.35, and large when >0.35 [30]. A 95% confidence level was used (*p* < 0.05). Data management and analysis were performed using the SPSS statistical package version 20 for the social sciences for Windows (SPSS Inc., Chicago, IL, USA).

## 3. Results

The characteristics of the participants of this study are displayed in Table 1. A total of 241 women (72.52 ± 3.93 years) finally took part in this study, 19.92% received secondary or university education and most of the women were retired (82.16%). The mean BMI indicated overweight, (28.49 kg/m2), the mean scores for HADS depression and anxiety were 8.83 ± 5.07 and 9.73 ± 6.46, respectively, while the PSQI total score was 6.17 ± 5.00, which indicates poor sleep quality. Mean scores in the MMSE (27.96 ± 2.26) and the MoCA (27.26 ± 2.34) test indicated normality.

The analysis of the individual associations showed that higher values of anxiety and depression, as well as well as poorer sleep quality in all the PSQI domains and the total score, were significantly related with worse scores in the MMSE [all *p* < 0.001, except for sleep latency (*p* = 0.003), sleep efficiency (*p* = 0.002), sleep disturbances (*p* = 0.028) and use of sleeping medication (*p* = 0.006)], MoCA (all *p* < 0.001, except for sleep disturbances (*p* = 0.002) and use of sleeping medication (*p* = 0.001), TMT-A (all *p* < 0.001) and TMT-B (all *p* < 0.001). The Isaacs test was associated with HADS depression (*p* < 0.001), sleep quality (*p* = 0.007), sleep latency (*p* = 0.043), use of sleeping medication (*p* = 0.007), daytime dysfunction (*p* < 0.001) and PSQI total score (*p* = 0.001), and the PSQI domains “sleep efficiency” and “sleep disturbances”. The analysis of the individual associations with the possible confounders showed that only higher age (Table 2) was related with poor results in MMSE (*p* = 0.001), MoCA (*p* < 0.001), and the Isaacs test (*p* = 0.019) scores. A partial correlation analysis controlled for age was performed (Table 3), and all the correlations remained except for those between sleep latency and verbal fluency, and between sleep disturbances and the MMSE score.

No significant results were observed regarding the education level or smoking habit (Table 4).

The independent associations between the cognitive capacities of the participants and sleep quality, anxiety and depression are displayed in Table 5. Elevated age and poor sleep quality regarding daytime dysfunction were independently associated with lower scores in the Isaacs test (worse verbal fluency), although size effect was small (adjusted R^2^ = 0.094). As for cognitive function, both elevated levels of anxiety and depression, as well as higher age, were independently related to lower MMSE scores and thus worse global cognitive function. The adjusted R^2^ was 0.306, which indicates a medium size effect. On the other hand, older age, higher anxiety levels and poor sleep quality due to daytime dysfunction were independently associated with a greater risk of developing mild cognitive impairment with a medium size effect (adjusted R^2^ = 0.248). Finally, as for executive function, worse sleep quality, represented by the PSQI domains sleep latency and sleep disturbances, as well as the total score, were independently associated with longer completion times in both TMT-A and TMT-B, which was also linked to more severe levels of anxiety symptoms. The effect sizes were large, with adjusted R^2^ values of 0.758 and 0.508 for TMT-A and TMT-B, respectively.

## 4. Discussion

The aim of this study was to evaluate the associations of sleep quality, anxiety, and depression with cognitive performance, executive functions, and verbal fluency among women aged 65 years and over. Our findings showed that poorer sleep quality was associated with worse cognitive performance and verbal fluency (PSQI daytime dysfunctions domain), and executive functions (PSQI total score, sleep latency domains, sleep disorders). Besides, both higher levels of anxiety and depression were associated with poor cognitive performance while only anxiety was linked to decreased executive functions. As for the possible confounders, age was associated with cognitive performance and verbal fluency.

Sleep problems have been shown to be associated with adverse physical and psychological health implications such as mood problems, physical and mental distress, and poor physical performance and activity limitations [31,32]. With regard to cognitive performance, previous studies have studied its association with sleep problems. Nebes et al. [33] indicated that deterioration in the normal structure of sleep in older adults can negatively affect cognitive performance, and the North Manhattan Study (NOMAS) have reported that long sleep duration is associated with poorer cognitive performance evaluated with the Mini-Mental State Examination among elderly community-dwelling adults [34]. This association may be due to the potential mechanism known as lack of recovery or restoration, since during sleep the rate of anabolism is at its peak and restoration occurs both in the central nervous system and throughout the body [35], so the interruption of sleep can affect this process. The results of the present study did not show independent associations of poor sleep quality and MMSE scores, which may be partly explained by the fact that the mean score and most of the participants (74.9%) were situated in the normal range. On the other hand, and regarding verbal fluency, a study has shown that sleep loss can affect the performance of this cognitive ability [36]. Similarly, the study by Ashley et al. [37] showed that specific components of sleep quality such as daytime dysfunction are associated with verbal fluency, but in female breast cancer survivors up to 10 years after chemotherapy. Our findings are in accordance with that and suggested that poor sleep quality related to daytime dysfunction was an independent predictor of greater cognitive impairment in community-dwelling older women.

Executive function is very important for public health, since it is a key factor for everyday functioning and preserving autonomy [38]. Adequate sleep with an optimal duration facilitates executive functions [39], but it has been suggested that sleep disturbance or sleep quality could be more important than sleeping time for executive function [38]. In this study, a relationship was found between sleep latency, sleep disturbances and the total score of the PSQI questionnaire with poor executive functions. To our knowledge, the association between the previously mentioned domains of the PSQI questionnaire and executive functions have not been reported to date. Lambiase et al. [38] observed that sleep efficiency measured by actigraphy was associated with executive function in older women, and a cross-sectional study with Spanish older people described an association between sleep duration with a decrease in cognitive functioning domains such as memory and executive function [40].

The risk of suffering anxiety and depression, two of the most commonly reported health-related problems in middle-aged women, increases after menopause onset [41]. Our findings showed that those women who had higher levels of anxiety and depression showed worse cognitive performance, as assessed with the MoCA and the MMSE tests, respectively. Menopause-related estrogen decline also increases the risk of cognitive impairment [42]. This finding is in agreement with those of Lindgren et al. [43], who found associations between affective symptoms and poorer cognitive performance, but unlike in our study, the correlations did not reach statistical significance. Similarly, previous studies have shown the association between anxiety and depression with cognitive performance in the general population [44,45], with some pathology such as Parkinson’s [46]. This may be because the association between cognitive performance and anxiety is shaped like an inverted U, so that higher cognitive performance is associated with intermediate levels of anxiety [47].

There is no consensus regarding the possible relationship between anxiety and depression with executive functions, which are the cognitive abilities that regulate behavior and encompass a series of cognitive elements such as working memory, cognitive flexibility or inhibition [48]. Associations between depression and executive function have been described, but in the general population [49,50], and Bunce et al. [51] did not find significant associations between anxiety and executive functions; however, they did demonstrate an association with depression. On the other hand, deficits in some components of executive function are frequently reported in adults with anxiety disorders [52]. Although the results of this study initially revealed individual associations of both greater anxiety and depressive symptoms with a longer time in the TMT-A and TMT-B tests, only anxiety remained related to the TMT-B in the linear regression analysis., while poor sleep quality outcomes remained as independent predictors with regard to the TMT-A.

This study has a number of limitations to be acknowledged. The cross-sectional design of the study does not allow causal relationships to be inferred. Although the PSQI questionnaire has been validated and used worldwide to assess sleep quality, objective measurement tools, such as accelerometry or polysomnography, were not used. Information on comorbidities (such as chronic illness or cancer), family relations, educational or economic status have not been included in the regression model. Finally, this study was carried out among women older than 65 years from a specific geographical area and any generalization of the findings should be limited to people with similar characteristics. Future studies should be performed on a more diverse target population that includes both men and women of different age groups, including other possible confounders, and using a prospective design and objective measures to assess sleep quality.

## 5. Conclusions

Among community-dwelling women aged 65 and over, and after considering possible confounders, sleep latency, sleep disturbances, and the total PSQI score were linked to decreased cognitive ability and executive function. On the other hand, poorer sleep quality, manifested as daytime dysfunction, was associated with worse cognitive performance and verbal fluency. Finally, higher levels of anxiety were related to lower executive functions and cognitive performance, while depression was only associated with the latter. Among the possible confounders, only age was found to be associated with cognitive performance and verbal fluency, and this should be considered when interpreting the results of the present study. Therefore, sleep quality, anxiety and depression should be taken into account in the management and prevention of cognitive function decline in community-dwelling women aged 65 years and over.

## Figures and Tables

**Table 1:** Descriptive data of the participants (*n* = 241).

Characteristics		Values	
		Mean	SD
Age (years)		72.52	3.93
Time since menopause (years)		23.09	6.59
		Frequency	Percentage
Education	Primary or less	193	80.08
	Secondary or superior	48	19.92
Ocupational status	Retired	198	82.16
	Worker	8	3.32
	unemployed	35	14.52
		Mean	SD
BMI		28.49	2.40
Anxiety (HADS)		9.73	6.46
Depression (HADS)		8.83	5.07
PSQI	Sleep quality	0.72	0,86
	Sleep latency	0.80	1.15
	Sleep duration	0.71	0.86
	Sleep efficiency	0.41	0.65
	Sleep disturbances	1.19	0.44
	Use of sleeping medication	1.04	1.18
	Daytime dysfunction	1.30	0.98
	Total score	6.17	5.00
MMSE		27.96	2.26
MoCA		27.26	2.34
Isaacs test		36.93	3.29
TMT-A (s)		87.33	43.28
TMT-B (s)		173.34	79.35

BMI: Body Mass Index. HADS: Hospital Anxiety and Depression Scale. MMSE: Mini Mental State Examination. MoCA: Montreal Cognitive Assessment. PSQI: Pittsburgh Sleep Quality Index. TMT-A: Trail Making Test—Part A. TMT-B: Trail Making Test—Part B.

**Table 2:** Pearson’s correlations between cognitive outcomes and anxiety, depression, sleep quality and possible confounders.

Variable			MMSE	MoCA	Isaacs Test	TMT-A	TMT-B
HADS Anxiety		r	−0.41	−0.35	−0.10	0.24	0.26
		*p*-value	<0.001	<0.001	0.130	<0.001	<0.001
HADS depression		r	−0.40	−0.34	−0.23	0.36	0.28
		*p*-value	<0.001	<0.001	<0.001	<0.001	<0.001
PSQI	Sleep quality	r	−0.25	−0.26	−0.17	0.68	0.52
		*p*-value	<0.001	<0.001	0.007	<0.001	<0.001
	Sleep latency	r	−0.19	−0.24	−0.13	0.83	0.68
		*p*-value	0.003	<0.001	0.043	<0.001	<0.001
	Sleep duration	r	−0.25	−0.27	−0.18	0.68	0.54
		*p*-value	<0.001	<0.001	0.005	<0.001	<0.001
	Sleep efficiency	r	−0.20	−0.26	−0.19	0.58	0.43
		*p*-value	0.002	<0.001	0.068	<0.001	<0.001
	Sleep disturbances	r	−0.14	−0.20	−0.09	0.68	0.57
		*p*-value	0.028	0.002	0.148	<0.001	<0.001
	Use of sleeping medication	r	−0.18	−0.21	−0.18	0.78	0.63
		*p*-value	0.006	0.001	0.007	<0.001	<0.001
	Daytime dysfunction	r	−0.40	−0.37	−0.29	0.58	0.46
		*p*-value	<0.001	<0.001	<0.001	<0.001	<0.001
	Total score	r	−0.29	−0.32	−0.21	0.86	0.68
		*p*-value	<0.001	<0.001	0.001	<0.001	<0.001
Age		r	−0.21	−0.24	−0.15	0.11	0.10
		*p*-value	0.001	<0.001	0.019	0.083	0.132
BMI		r	−0.11	−0.06	−0.05	−0.02	0.02
		*p*-value	0.102	0.353	0.407	0.725	0.793
Years since menopause onset		r	−0.04	−0.00	−0.03	0.03	0.01
		*p*-value	0.522	0.955	0.664	0.645	0.885
Minutes of exercise/day		r	−0.01	0.03	−0.03	−0.07	−0.01
		*p*-value	0.831	0.597	0.621	0.263	0.903

BMI: Body Mass Index. HADS: Hospital Anxiety and Depression Scale. MMSE: Mini Mental State Examination. MoCA: Montreal Cognitive Assessment. PSQI: Pittsburgh Sleep Quality Index. TMT-A: Trail Making Test—Part A. TMT-B: Trail Making Test—Part B.

**Table 3:** Partial correlations between cognitive outcomes and anxiety, depression, sleep quality controlled for age.

Variable			MMSE	MoCA	Isaacs Test	TMT-A	TMT-B
HADS Anxiety		r	−0.40	−0.34	−0.09	0.23	0.26
		*p*-value	<0.001	<0.001	0.172	<0.001	<0.001
HADS depression		r	−0.39	−0.33	−0.22	0.36	0.28
		*p*-value	<0.001	<0.001	<0.001	<0.001	<0.001
PSQI	Sleep quality	r	−0.23	−0.24	−0.16	0.67	0.52
		*p*-value	<0.001	<0.001	0.016	<0.001	<0.001
	Sleep latency	r	−0.18	−0.23	−0.12	0.82	0.68
		*p*-value	0.005	<0.001	0.063	<0.001	<0.001
	Sleep duration	r	−0.23	−0.25	−0.16	0.68	0.53
		*p*-value	<0.001	<0.001	0.012	<0.001	<0.001
	Sleep efficiency	r	−0.18	−0.24	−0.10	0.56	0.42
		*p*-value	0.005	<0.001	0.119	<0.001	<0.001
	Sleep disturbances	r	−0.12	−0.18	−0.08	0.68	0.56
		*p*-value	0.063	0.005	0.232	<0.001	<0.001
	Use of sleeping medication	r	−0.16	−0.18	−0.16	0.77	0.63
		*p*-value	0.016	0.006	0.015	<0.001	<0.001
	Daytime dysfunction	r	−0.39	−0.36	−0.28	0.58	0.45
		*p*-value	<0.001	<0.001	<0.001	<0.001	<0.001
	Total score	r	−0.27	−0.30	−0.20	0.85	0.68
		*p*-value	<0.001	<0.001	0.002	<0.001	<0.001

HADS: Hospital Anxiety and Depression Scale. MMSE: Mini Mental State Examination. MoCA: Montreal Cognitive Assessment. PSQI: Pittsburgh Sleep Quality Index. TMT-A: Trail Making Test—Part A. TMT-B: Trail Making Test—Part B.

**Table 4:** Individual differences regarding smoking habit and education.

Variable	Smokers					Education				
	No (*n* = 213)		Yes (*n* = 28)			Primary or less (*n* = 193)		Secondary or higher (*n* = 48)		
	Mean	DT	Mean	DT	*p*-value	Mean	DT	Mean	DT	*p*-value
MMSE	27.94	2.28	28.11	2.11	0.712	27.87	2.33	28.33	1.93	0.200
MoCA	27.27	2.38	27.21	2.08	0.910	27.21	2.37	27.46	2.26	0.517
Isaacs test	36.99	3.26	36.50	3.57	0.464	36.82	3.36	37.35	3.02	0.319
TMT-A	87.49	44.05	86.11	37.53	0.874	88.16	42.50	84.00	46.59	0.552
TMT-B	172.95	79.05	176.36	83.08	0.831	175.64	78.99	164.13	80.98	0.369

MMSE: Mini Mental State Examination. MoCA: Montreal Cognitive Assessment. TMT-A: Trail Making Test—Part A. TMT-B: Trail Making Test—Part B.

**Table 5:** Multivariate linear regression analyses.

Variable		B	Beta	t	95% CI		*p*-Value
MMSE	HADS anxiety	−0.13	−0.36	−6.62	−0.16	−0.09	<0.001
	HADS depression	−0.15	−0.34	−6.28	−0.20	−0.10	<0.001
	Age	−0.09	−0.16	−2.90	−0.15	−0.03	0.004
MoCA	Daytime dysfunction	−0.74	−0.31	−5.45	−1.01	−0.48	<0.001
	HADS anxiety	−0.10	−0.29	−5.07	−0.15	−0.06	<0.001
	Age	−0.11	−0.19	−3.28	−0.18	−0.04	0.001
Isaacs test	Daytime dysfunction	−0.95	−0.28	−4.55	−1.36	−0.54	<0.000
	Age	−0.10	−0.12	−2.00	−0.21	0.00	0.047
TMT-A	PSQI total score	4.38	0.50	7.11	3.16	5.59	<0.001
	Sleep latency	10.75	0.29	4.14	5.63	15.87	<0.001
	Sleep disturbances	13.44	0.14	3.06	4.79	22.09	0.002
TMT-B	PSQI total score	4.75	0.30	2.93	1.55	7.94	0.004
	Sleep latency	20.68	0.30	3.03	7.24	34.12	0.003
	HADS anxiety	1.33	0.11	2.32	0.20	2.46	0.021
	Sleep disturbances	25.27	0.14	2.19	2.58	47.97	0.029

B: unstandardized coefficient. Beta: standardized coefficient. CI: confidence interval. HADS: Hospital Anxiety and Depression Scale. MMSE: Mini Mental State Examination. MoCA: Montreal Cognitive Assessment. PSQI: Pittsburgh Sleep Quality Index. TMT-A: Trail Making Test—Part A. TMT-B: Trail Making Test—Part B.

## Data Availability

The data shown in this study are available upon request from the corresponding author. The data is not available to the public, since taking into account the sensitive nature of all the questions asked in this study, all participants were guaranteed that the data obtained would be confidential and would not be shared.

## References

[1] Alvarado A.M., Salazar A.M. (2014). Aging concept analysis. Gerokomos.

[2] (2014). Estadísticas Mundiales: Una Mina de Información Sobre Salud Pública Mundial.

[3] Luarte C., Poblete F., Flores C., Duarte F. (2016). Physical Parameters, cognition and its relationship with the quality of life in elderly Talcahuano, Concepción, Valdivia y Osorno. Revista Ciencias de la Actividad Física.

[4] Shirvani M., Heidari M. (2016). Quality of Life in Postmenopausal Female Members and Non-members of the Elderly Support Association. J. Menopausal Med..

[5] Pawlak I.E., Wolinska W., Mroczek B. (2016). Impact of climacteric and depressive symptoms on the quality of life of postmenopausal women. Fam. Med. Prim. Care Rev..

[6] Chowdhury E.K., Berk M., Nelson M.R., Wing L.M., Reig C.M. (2019). Association of depression with mortality in an elderly treated hypertensive population. Int. Spychogeriat..

[7] Valencia P.D. (2019). The Depression Anxiety Stress Scales (DASS-21): Do they measure anything beyond a general factor?. Avances en Psicologia.

[8] Centikol G., Bastug G., Ozel E.T. (2020). Poor Acceptance of the Past is Related to Depressive Symptoms in Older Adults. GeroPsych.

[9] Briant C., Jackson H., Ames D. (2008). The prevalence of anxiety in older adults. J. Affect. Disord..

[10] Zisberg A. (2017). Anxiety and depression in older patients: The role of culture and acculturation. Int. J. Equity Heath.

[11] Rubio J.A., Rodríguez R., Andreu L., Martínez L.M., Martínez A., Ramos D.J. (2019). Effect of Sleep Quality on the Prevalence of Sarcopenia in Older Adults: A Systematic Review with Meta-Analysis. J. Clin. Med..

[12] Dzierzewski J.M., Dautovich N., Ravyts S. (2018). Sleep and Cognition in Older Adults. Sleep Med Clin..

[13] Reid K., Krauchi K., Grimaldi D., Sbarboro J., Attarian H., Malkani R., Matteo M., Cee P.C. (2021). Effects of manipulating body temperature on sleep in postmenopausal women. Sleep Med..

[14] Sutter C., Zollig G., Allemand M., Martin M. (2012). Sleep Quality and Cognitive Function in Healthy Old Age: The Moderating Role of Subclinical Depression. Neurospsychiology.

[15] López A.G., Calero M.D. (2009). Predictors of cognitive decline in the elderly. Rev. Esp. Geriatr. Gerontol..

[16] Bojar I., Pinkas J., Gujski M., Owoc A., Raczkiewicz D., Gustaw-Rothenberg K. (2017). Postmenopausal cognitive changes and androgen levels in the context of apolipoprotein E polymorphism. Arch. Med. Sci..

[17] Scheyer O., Rahman A., Hristov H., Bercowitz C., Iaacson R.S., Diaz R., Mosconi L. (2018). Female Sex and Alzheimer’s Risk: The Menopause Connection. J. Prev. Alzheimer Dis..

[18] Evans H.M., Howe P.R., Wong R.H. (2017). Effects of Resveratrol on Cognitive Performance, Mood and Cerebrovascular Function in Post-MenopausalWomen; A 14-Week Randomised Placebo-Controlled Intervention Trial. Nutrients.

[19] World Health Organization (2000). Obesity: Preventing and Management of the Global Epidemic. Report of the WHO Consultation. Technical Report Series. No. 894.

[20] Zigmond A.S., Snaith R.P. (1983). The hospital anxiety and depression scale. Acta. Psychiatr. Scand..

[21] Herrero M.J., Blanch J., Peri J.M., De Pablo J., Pintor L., Bulbena A. (2003). A validation study of the hospital anxiety and depression scale (HADS) in a Spanish population. Gen. Hosp. Psychiatr..

[22] Buysse D.J., Reynolds C.F., Monk T.H., Berman S.R., Kupfer D.J. (1989). The Pittsburgh Sleep Quality Index: A new instrument for psychiatric practice and research. Psychiatry Res..

[23] Hita-Contreras F., Martínez-López E., Latorre-Román P.A., Garrido F., Santos M.A., Martínez-Amat A. (2014). Reliability and validity of the Spanish version of the Pittsburgh Sleep Quality Index (PSQI) in patients with fibromyalgia. Rheumatol. Int..

[24] Doi Y., Minowa M., Uchiyama M., Okawa M., Kim K., Shibui K., Kamei Y. (2000). Psychometric assessment of subjective sleep quality using the Japanese version of the Pittsburgh Sleep Quality Index (PSQI-J) in psychiatric disordered and control subjects. Psychiatr. Res..

[25] Lobo A., Saz P., Marcos G., Día J.L., de la Cámara C., Ventura T., Morales-Asín F., Fernando-Pascual L., Montañés J.A., Aznar S. (1999). Revalidation and standardization of the cognition mini-exam (first Spanish version of the Mini-Mental Status Examination) in the general geriatric population. Med. Clin..

[26] Nasreddine Z.S., Phillips N.A., Bedirian V., Charbonneau S., Whitehead V., Collin I., Cummings J.L., Chertkow H. (2005). The Montreal cognitive assessment, MoCA: A brief screening tool for mild cognitive impairment. J. Am. Geriatr. Soc..

[27] Isaacs B., Kennie A.T. (1971). The Set Test as an Aid to the Detection of Dementia in Old People. Br. J. Psychiat..

[28] Reitan R.M. (1958). Trail Making Test: Manual for Administration, Scoring and Interpretation.

[29] Concato J., Peduzzi P., Holford T.R., Feinstein A.R. (1995). Importance of events per independent variable in proportional hazards analysis. I. Background, goals, and general strategy. J. Clin. Epidemiol..

[30] Cohen J.A. (1992). A power primer. Psychol. Bull..

[31] Strine T.W., Chapman D.P. (2005). Associations of frequent sleep insufficiency with health-related quality of life and health behaviors. Sleep Med..

[32] Denison H.J., Jameson K.A., Sayer A.A., Patel H.P., Edwards M.H., Arora T., Dennison E.M., Cooper C., Baird J. (2021). Poor sleep quality and physical performance in older adults. Sleep Health.

[33] Nebes R.D., Buysse D.J., Halligan E.M., Houck P.R., Monk T.H. (2009). Self-reported sleep quality predicts poor cognitive performance in healthy older adults. J. Gerontol. B Psychol. Sci. Soc. Sci..

[34] Ramos A.R., Dong C., Elkind M.S., Boden B., Sacco R.L., Rundek T., Wrihte C.B. (2013). Association between sleep duration and the mini-mental score: The Northern Manhattan study. J. Clin. Sleep Med..

[35] Blackwell T., Yaffe K., Laffan A., Ancoli-Israel S., Redline S., Ensrud K.E., Song Y., Stone K.L., Osteoporotic Fractures in Men (MrOS) Study Group (2014). Associations of objectively and subjectively measured sleep quality with subsequent cognitive decline in older community-dwelling men: The MrOS sleep study. Sleep.

[36] Horne J.A. (1988). Sleep loss and "divergent" thinking ability. Sleep.

[37] Henneghan A.M., Carter P., Stuifbergan A., Parmelee B., Kesler S. (2018). Relationships between self-reported sleep quality components and cognitive functioning in breast cancer survivors up to 10 years following chemotherapy. Psychooncology.

[38] Lambiase M.J., Gabriel K.P., Kuller L.H., Matthews K.A. (2014). Sleep and executive function in older women: The moderating effect of physical activity. J. Gerontol. A Biol. Sci. Med. Sci..

[39] Vyazovskiy V.V. (2015). Sleep, recovery, and metaregulation: Explaining the benefits of sleep. Nat. Sci. Sleep.

[40] Faubel R., López-García E., Guallar-Castillón P., Graciani A., Banegas J.R., Rodríguez-Artalejo F. (2009). Usual sleep duration and cognitive function in older adults in Spain. J. Sleep Res..

[41] Tangen T., Mykletun A. (2008). Depression and anxiety through the climacteric period: An epidemiological study (HUNT-II). J. Psychosom. Obstet. Gynaecol..

[42] Russell J.K., Jones C.K., Newhouse P.A. (2019). The role of estrogen in brain and cognitive aging. Neurotherapeutics.

[43] Lindgren M., Birling H., Kieseppä T., Tuulio-Henriksson A. (2020). Is cognitive performance associated with anxiety and depression in first-episode psychosis?. J. Affect. Disord..

[44] Castaneda A.E., Tuulio-Henriksson A., Marttunen M., Suvisaari J., Lönnqvist J. (2008). A review on cognitive impairments in depressive and anxiety disorders with a focus on young adults. J. Affect. Disord..

[45] Eysenck M.W., Derakshan N., Santos R., Calvo M.G. (2007). Anxiety and cognitive performance: Attentional control theory. Emotion.

[46] Petkus A.J., Filoteo J.V., Schiehser D.M., Gomez M.E., Petzinger G. (2019). Worse cognitive performance predicts increased anxiety and depressive symptoms in patients with Parkinson’s disease: A bidirectional analysis. Neuropsychology.

[47] Salthouse T.A. (2012). How general are the effects of trait anxiety and depressive symptoms on cognitive functioning?. Emotion.

[48] Miyake A., Friedman N.P. (2012). The Nature and Organization of Individual Differences in Executive Functions: Four General Conclusions. Curr. Dir. Psychol. Sci..

[49] Franz C.E., Lyons M.J., O’Brien R., Panizzon M.S., Kim K., Bhat R., Grant M.D., Toomey R., Eisen S., Xian H. (2011). A 35-year longitudinal assessment of cognition and midlife depression symptoms: The Vietnam Era Twin Study of Aging. Am. J. Geriatr. Psychiatry.

[50] Holmes A.J., Pizzagalli D.A. (2007). Task feedback effects on conflict monitoring and executive control: Relationship to subclinical measures of depression. Emotion.

[51] Bunce D., Handley R., Gaines S.O. (2008). Depression, anxiety, and within-person variability in adults aged 18 to 85 years. Psychol. Aging.

[52] Zebdi R., Goyet L., Pinabiaux C., Guellaï B. (2016). Psychological Disorders and Ecological Factors Affect the Front Development of Executive Functions: Some Perspectives. Front. Psychiatry.

